# Mediterranean Diet Patterns in Relation to Lung Cancer Risk: A Meta-Analysis

**DOI:** 10.3389/fnut.2022.844382

**Published:** 2022-04-11

**Authors:** Hongzhen Du, Tengrui Cao, Xuning Lu, Tianfeng Zhang, Bin Luo, Zengning Li

**Affiliations:** ^1^Department of Nutrition, The First Hospital of Hebei Medical University, Shijiazhuang, China; ^2^Hebei Provincial Key Laboratory of Nutrition and Health, Shijiazhuang, China

**Keywords:** Mediterranean diet, lung cancer, risk of occurrence, meta-analysis, dose–response

## Abstract

**Background:**

We systematically quantified the currently inconclusive association between Mediterranean diet patterns and the risk of lung cancer.

**Methods:**

We searched the PubMed, Cochrane, Database of Abstracts of Reviews of Effects (DARE) and Web of Science electronic databases to identify relevant articles published before October 2021. We used the Newcastle–Ottawa scale to assess the quality of the published research and a random-effects model to estimate the aggregate hazard ratios and 95% CIs. As a result of significant heterogeneity, we performed subgroup analysis, meta-regression analysis, and sensitivity analysis. Where data were available, we also performed a dose–response analysis.

**Results:**

Nine articles were included in the meta-analysis. The meta-analysis showed that there was a significant negative correlation between Mediterranean diet patterns and the risk of lung cancer in the general population with a hazard ratio of 0.82, a 95% CI of 0.74–0.92, and a high heterogeneity (*I*^2^ = 59.9%, *P* < 0.05). As a result of the significant heterogeneity, we conducted subgroup analysis, meta-regression analysis, and sensitivity analysis and found that the study design was the source of the heterogeneity. Subgroup analysis and sensitivity analysis showed that the final results did not change very much, the sensitivity was low and the results were relatively stable. The dose–response relationship showed that, based on the lowest Mediterranean diet score (0 points), for every three-point increase, the risk of lung cancer was reduced by 9%.

**Conclusion:**

The evidence in this meta-analysis shows that there is a significant negative correlation between Mediterranean diet patterns and the risk of lung cancer, suggesting that Mediterranean diets are a protective factor in lung cancer.

## Introduction

Lung cancer has become the top malignant tumor in terms of morbidity and mortality (1). Lung cancer primarily originates from lung epithelial cells and is caused by epithelial cell lesions, which lead to uncontrolled cell proliferation. Severe lung cancer will metastasize and invade the thoracic cavity, hilar lymph nodes, and distant organs, namely, the brain, liver, and kidneys. The factors affecting the occurrence, development, and metastasis of lung cancer are diverse and complex but can be roughly divided into factors related to the tumor cells and the tumor microenvironment. The microenvironment of the tumor includes the immune and non-immune microenvironments, which significantly affect the occurrence and development of lung cancer. Our current understanding of the molecular mechanisms of the occurrence, development, metastasis, and drug resistance of lung cancer is still limited. Established risk factors include genetics, the environment, and lifestyle. Although cigarette smoking is still a major cause of lung cancer, a considerable proportion of patients who develop lung cancer do not have a significant personal history of tobacco use (2). The higher incidence of lung cancer and mortality from this disease among Chinese women is related to both the outdoor air pollution and the indoor use of solid fuels (3, 4). We, therefore, need to explore the factors that are protective for lung cancer to reduce its incidence and improve the prevention and treatment of this disease.

The role of dietary nutrients in the prevention and treatment of lung cancer has become a focus of recent research. Vieira et al. (5) showed that high intakes of fruit and vegetables can significantly decrease the occurrence of lung cancer. Some studies have shown that high intakes of cholesterol are positively correlated with the incidence of lung cancer (6). The long-term, high-dose use of supplements containing β-carotene, retinyl palmitate, and vitamins E, B, B6, and B12 for patients with lung cancer needs to be advised with caution (7). The results of a meta-analysis of articles showed that healthy dietary patterns are associated with a lower risk of lung cancer (8). Combining this strong evidence with the healthy eating patterns proposed in emerging medical prescriptions suggests that a Mediterranean diet pattern is a good model of healthy eating (9).

Mediterranean diets are sustainable dietary patterns with the following characteristics: (a) a high intake of cereals, vegetables, fruits, beans, and nuts; (b) a moderate intake of poultry, fish, eggs, fresh seafood, and dairy products and a regular, moderate intake of wine; (c) a low intake of red meat, processed meat, and sugary foods; and (d) the use of vegetable oils containing unsaturated fatty acids instead of animal fats containing saturated fatty acids when cooking, especially olive oil (10). Epidemiological studies have recently begun to pay attention to the relationship between Mediterranean diets and the risk of lung cancer.

Gnagnarella et al. (11) showed that adhering to a Mediterranean diet pattern may reduce the incidence of lung cancer. A 2016 collaborative cohort study also concluded that, among smokers, adherence to the Mediterranean diet was significantly negatively correlated with the risk of lung cancer (12). A multiethnic cohort study of 179,318 Americans aged 45–75 years in 2021 emphasized that a Mediterranean diet was associated with a low risk of lung cancer (13). However, some studies have not found a significant association between Mediterranean diet patterns and lung cancer. For example, a cohort study from Iran that included 48,421 participants concluded that a Mediterranean diet pattern had no association with the risk of lung cancer (14). Similarly, a cohort study by Schulpen et al. in the Netherlands showed no significant correlation between Mediterranean diets and the risk of lung cancer (15).

The relationship between Mediterranean diets and the risk of lung cancer is still controversial. We, therefore, need to conduct a comprehensive meta-analysis to investigate this relationship. This research aimed to systematically study and review observational articles to determine the relationship between Mediterranean diet patterns and lung cancer. Through this meta-analysis, we aim to understand the influence of Mediterranean diet patterns on the health of the general population, improve quality of life, and explore the protective factors of this diet pattern toward lung cancer to provide a theoretical basis for reducing the incidence of this disease.

## Materials and Methods

### Search Strategy

We searched all the studies included in the PubMed, Cochrane, Database of Abstracts of Reviews of Effects (DARE), and Web of Science electronic databases before October 2021. The following search terms were used: (“diet Mediterranean” or “Mediterranean diet” or “diets Mediterranean” or “Mediterranean diets”) and (“lung neoplasms” or “pulmonary neoplasms” or “neoplasms lung” or “lung neoplasm” or “neoplasms pulmonary” or “neoplasm pulmonary” or “pulmonary neoplasm” or “lung cancer” or “cancer lung” or “cancers lung” or “lung cancers” or “pulmonary cancer” or “cancer pulmonary” or “cancers pulmonary” or “pulmonary cancers” or “cancer of the lung” or “cancer of lung”). We also searched the references of related articles to obtain other potential studies.

### Study Selection

We screened the articles based on their titles and abstracts. We excluded studies that did not include a correlation between the Mediterranean diet and lung cancer. We used the following inclusion criteria: (a) the incidence of lung cancer was used as the outcome; (b) the study design was case–control, cohort, and follow-up studies of randomized clinical trials; and (c) the study provided the odds ratio (OR), risk ratio (RR), hazard ratio (HR) of lung cancer, and the 95% CI, or sufficient data to fully estimate these values. We excluded review articles, studies not published as a full article (e.g., conference abstracts) and studies lacking sufficient data relevant to lung cancer. For overlapping publications, we selected the most recent data with the longest follow-up time.

### Data Extraction and Quality Assessment

The data were extracted independently by two investigators (HZD and TRC) from studies that met the inclusion criteria and any ambiguities were resolved by a third investigator. We extracted the following information from each study: first author; year of publication; country; study type; duration of the study; sample size; age; sex; the number of participants in different groups; Mediterranean diet score (MDS) system; components of the MDS; risk estimates; the 95% CIs from the most fully adjusted models for the association between a Mediterranean diet and lung cancer; and adjustment factors.

We evaluated the methodological quality of the included studies according to the Newcastle–Ottawa scale (NOS) (16). The total NOS score was nine points. Studies identified as having the NOS ≥ 7 are considered high quality, whereas studies with a total NOS score < 7 are considered low quality. All the included studies were of high quality.

### Definition of the Mediterranean Diet Score

The MDS was defined specifically to measure adherence to the Mediterranean diet. In our meta-analysis, a Mediterranean diet alternative score (aMED) was adapted for five studies (13, 14, 17–19). Two studies used the Polish MDS (Polish-aMED) (20, 21), one used the MDS (12) and one used both the modified MDS (mMED) and the aMED (15). The MDS provides 0 or 1 point for each component. The MDS ranged from 0 to 9 or from 0 to 8 depending on whether alcohol was included. Table 1 gives more details about the components of the MDS.

**TABLE 1:** Characteristics of studies entered in the meta-analysis.

References	Country	Study type	Time	Sex	Population, n	Median age	Md score system and components of MD score	MD scores in each group	The cases and participants in each group	HR/OR (95%CI) comparison level	Adjustment variables
Maisonneuve et al. (17)	Italy	Cohort study	2004–2005	F/M	F:1,468 M:2,868	57.3	aMED (1) vegetables (2) legumes (3) fruits (4) nuts (5) whole-grain cereals (6) fish (7) fatty acid ratio (MUFA:SFA ratio) (8) red and processed meats (9) alcohol	0–1	16/224	Ref	Age, gender, duration of smoking, daily average cigarette consumption, years of smoking cessation, asbestos exposure, total energy intake
								2–4	110/2,159	0.71 (0.42, 1.21)	
								5–7	72/1,795	0.59 (0.34, 1.02)	
								8–9	2/158	0.19 (0.04, 0.83)	
Hodgeet al. (12)	Australia	Cohort study	1990–1994	F/M	F:21,237 M:14,066	54.7	MDS (1) vegetables (2) fruit (3) cereals (4) legumes (5) fish (6) dairy (7) red meat (not chicken) (8) alcohol (9) olive oil	0–3	126/7,633	Ref	Cigarette pack years, smoking cessation years, smoking status, country of birth, education level, body mass index, physical activity, gender, energy (including the interaction between smoking status and country of birth)
								4–6	229/22,242	0.75 (0.60, 0.94)	
								7–9	48/5,428	0.64 (0.45, 0.90)	
Anic et al. (18)	America	Cohort study	1995–2006	F/M	F:18,3596 M:277,174	61.8	aMED (1) vegetables (2) legumes (3) fruits (4) nuts (5) whole-grain cereals (6) fish (7) fatty acid ratio (MUFA:SFA ratio) (8) red and processed meats (9) alcohol	NR	NR	Q5 vs. Q1: 0.85 (0.79, 0.91)	Age, gender, race, education level, body mass index, physical activity, total energy, smoking status, daily smoking amount, time to quit smoking and regular use of cigar/pipe
Schulpen et al. (15)	Netherlands	Cohort study	1986–2016	F/M	F;62,573 M:58,279	61.0	aMED (1) vegetables (2) legumes (3) fruits (4) nuts (5) whole-grain cereals (6) fish (7) fatty acid ratio (MUFA:SFA ratio) (8) red and processed meats	0–3	M: 1,153/11,842	Ref	Daily energy intake, alcohol consumption, body mass index, non-professional physical activity, highest level of education, family history of lung cancer, and history of chronic bronchitis diagnosed by a doctor
								4–5	970/12,579	0.86 (0.73, 1.02)	
								6–8	F: 290/4,784	0.91 (0.72, 1.15)	
								0–3	209/12,244	Ref	
								4–5	187/15,116	0.87 (0.65, 1.15)	
								6–8	52/6,279	0.73 (0.49, 1.09)	
							mMED (1) vegetables (2) legumes (3) fruits (4) nuts (5) whole and refined grains (6) fish (7) MUFA + PUFA:SFA ratio (8) total meat	0–3	M: 941/10,876	Ref	
								4–5	1,131/13,539	1.11 (0.93, 1.32)	
								6–8	341/4,791	0.96 (0.76, 1.21)	
								0–3	F: 188/11,653	Ref	
								4–5	207/16,514	0.99 (0.75, 1.32)	
								6–8	53/5,473	0.83 (0.56, 1.24)	
Krusinska et al. (20)	Poland	Case–control study	2013–2016	F/M	F:280 M:280	60.9	Polish-aMED (1) vegetables (2) fruit (3) whole grains (4) fish (5) legumes (6) nuts and seeds (7) the ratio of vegetables oils to animal fat (8) red and processed meats	0–2	75/113	Ref	Age, gender, cancer type, body mass index, socioeconomic status, general physical activity, smoking status, and alcohol abuse
								3–5	136/278	0.49 (0.30, 0.80)	
								6–8	69/169	0.37 (0.21, 0.64)	
Hawrysz et al. (21) a	Poland	Case–control study	2013–2017	M	M:439	62.6	Polish-aMED (1) vegetables (2) fruit (3) whole grains (4) fish (5) legumes (6) nuts and seeds (7) the ratio of vegetables oils to animal fat (8) red and processed meats (9) alcohol	0–3	97/177	Ref	Age, body mass index, current smoking status, socioeconomic status, general physical activity, incidence of lung cancer in relatives, and occupational exposure in the workplace
								3–5	83/221	0.51 (0.31, 0.81)	
								7–9	7/41	0.51 (0.32, 0.81)	
Park et al. (13)	America	Cohort study	1993–2014	F/M	F:97,699 M:81,619	59.9	aMED (1) vegetables (2) legumes (3) fruits (4) nuts (5) whole-grain cereals (6) fish (7) fatty acid ratio (MUFA:SFA ratio) (8) red and processed meats (9) alcohol	0–2	1,362/35,864	Ref	Age, gender, race/ethnicity, family history of lung cancer, education, body mass index, physical activity and total energy intake time indicators, including smoking status, average daily smoking, square of average daily smoking, number of years after smoking, smoking Interaction between number of years, race/ethnicity and smoking status, average number of cigarettes per day, square of average number of cigarettes per day, and number of years of smoking
								3	1,063/35,864	0.94 (0.86, 1.02)	
								4	988/35,864	0.86 (0.79, 0.94)	
								5	846/35,864	0.83 (0.75, 0.91)	
								6–9	1,091/35,86	0.83 (0.76, 0.91)	
Wang et al. (14)	Iran	Cohort study	2004–2008	F/M	F:28,016 M:20,372	52.0	aMED (1) vegetables (2) legumes (3) fruits (4) nuts (5) whole-grain cereals (6) fish (7) fatty acid ratio (MUFA:SFA ratio) (8) red and processed meats (9) alcohol	NR	NR	T3 vs. 1: 1.40 (0.84, 2.32)	Gender, age, race, body mass index, education level, place of residence, socioeconomic score, marital status, opium use, alcohol consumption, total energy intake, and physical activity
Myneni et al. (19)	America	Cohort study	1993–1998	F	F:86,090	63.6	aMED (1) vegetables (2) legumes (3) fruits (4) nuts (5) whole-grain cereals (6) fish (7) fatty acid ratio (MUFA:SFA ratio) (8) red and processed meats (9) alcohol	NR	NR	Q5 vs. 1: 0.96 (0.81, 1.13)	Age, race, education level, body mass index, physical activity, active smoking, years of exposure to second-hand smoke during childhood and adulthood, and energy intake

*aMED, Mediterranean diet alternative score; MDS, Mediterranean Diet Score; mMED, Modified Mediterranean Diet score; Polish-aMED, Adaptation to the Polish Mediterranean diet score; MUFA, monounsaturated fatty acid; PUFA, polyunsaturated fatty acid; SFA, saturated fatty acid; OR, odds ratio; HR, hazard ratio; NR, not reported.*

### Statistical Analysis

This meta-analysis aimed to examine the relationship between the Mediterranean diet and the risk of lung cancer in the general population. The associated effects include the OR and HR. Considering that lung cancer is a rare disease with an incidence rate of < 5/1,000, the OR and HR values can be directly combined with the effect size and expressed by the HR value. The most fully adjusted HRs with their 95% CIs from individual studies were extracted and transformed to their logarithms to stabilize the variances and normalize the distributions.

*I*^2^ and *P*-values were used to test the heterogeneity of the included studies. *P* < 0.05 was considered heterogeneous. *I*^2^ > 50% indicated high heterogeneity, 25–50% indicated moderate heterogeneity, and *I*^2^ < 25% indicated low heterogeneity. A fixed-effects model was used for combined analysis. Otherwise, a random-effects model was used for analysis when no significant heterogeneity (*I*^2^ < 50% or *P* > 0.05) was observed. As a result of the heterogeneity of the results, we conducted subgroup analysis based on several variables, namely, geographical area, study type, and other study characteristics. We also used meta-regression to explore the source of the heterogeneity. We used Begg’s test (22) and Egger’s regression asymmetry test (23) to test the publication bias and present them in the form of a funnel chart. We conducted a sensitivity analysis in which the meta-analysis was repeatedly carried out after omitting each study in turn to observe the stability of the comprehensive results.

The method for determining the dose–response relationship in the meta-analysis was the same as described previously (24). We needed to judge whether this was a linear relationship and we, therefore, conducted a non-linear dose–response meta-analysis using restricted cubic spline models. The results showed a linear dose–response relationship with *P* > 0.05. We then performed a linear dose–response meta-analysis using the glst function and plotted a linear dose–response relationship diagram. For studies that did not report the number of cases or person-years for each exposure category, the data were estimated by multiplying the proportion of cases in each category by the total person-years using a previously reported method (25). The median or mean values of the MDS for each category were obtained from the original studies when available. When the median or mean values per category were not reported, we calculated the midpoint of the upper and lower boundaries in each category. When the category was open-ended, we used the width of the adjacent interval as the width of the interval. All the analyses were performed using Stata version 11.0.

## Results

### Literature Search

**FIGURE 1:**
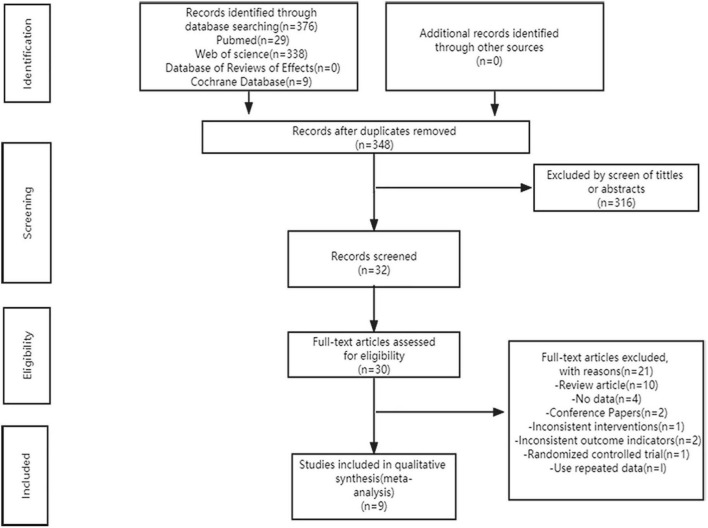
The PRISMA flow diagram of the literature search and study selection.

Figure 1 shows our systematic search of the literature. According to the search strategy, we obtained 376 articles from the databases and 348 articles remained after excluding duplicates. Subsequently, 316 articles were excluded by screening the titles and abstracts and the 32 remaining articles were evaluated further. Based on the inclusion and exclusion criteria, we excluded 23 articles, of which two articles had no full text, ten were reviews, four had no complete data, two were conference papers, one did not meet the intervention measures, two did not meet the outcome indicators, one was a randomized controlled trial, and one used repeated data. The remaining nine high-quality articles were included in the meta-analysis (12–15, 17–21).

### Research Characteristics

The nine articles included two case–control studies and seven cohort studies, the main characteristics of which are shown in Table 1. These studies were published between 2016 and 2021. Three studies were conducted in the United States, four studies were conducted in Europe, one study was conducted in Australia, and one study was conducted in Iran. The sample size ranged from 439 to 460,770 cases (936,089 cases in total). Seven studies included both the men and women, one study included men only, and one study included women only. All the nine studies reported the risk of lung cancer associated with Mediterranean diet patterns and the results were evaluated based on various sources, namely, medical records, cancer registries, and hospital databases. Table 2 shows the quality evaluation results. The NOS score ranged from 7 to 9 with an average of 8.

**TABLE 2:** Methodological quality of studies included in the meta-analysis.

Cohort studies^a^	Selection				Comparability^b^	Outcome			Score
	Representativeness of the exposed cohort	Selection of the unexposed cohort	Ascertainment of exposure	Outcome of interest not present at start of study	Control for important factor or additional factor	Outcome assessment	Follow-up long enough for outcome to occur^c^	Adequacy of follow-up of cohorts^d^	
Maisonneuve (17)	*	*	*	*	**	*	*		8
Hodge (12)	*	*	*	*	*	*	*		7
Anic (18)	*	*	*	*	**	*	*		8
Schulpen (15)	*	*	*	*	**	*	*	*	9
Park (13)	*	*	*	*	**	*	*		8
Wang (14)	*	*	*	*	**	*	*	*	9
Myneni (19)	*	*	*	*	**	*	*	*	9

**Case-control studies^a^**	**Selection**				**Comparability^b^**	**Exposure**			**Score**
	**Case definition**	**Representativeness**	**Control selection**	**Control definition**	**Control for important factor or additional factor**	**Ascertainment of exposure**	**Same method of ascertainment for cases and controls**	**Non-response rate^e^**	

Krusinska et al. (20)	*	*	*	*	**	*	*		8
Hawrysz et al. (21)	*	*	*	*	**	*	*		8

*^a^A study can be awarded a maximum of one star for each item except the item “Comparability.”*

*^b^A maximum of two stars can be awarded for this item. Studies controlling for or matching by age, gender received one star while studies additionally controlling for other important confounders received an additional star.*

*^c^A cohort study with a follow-up time of more than 5 years was assigned one star.*

*^d^A cohort study with a follow-up rate of more than 90% was assigned one star.*

*^e^One star was assigned if there was no statistically significant difference in the response rate between controls and cases using a chi-square test (P > 0.05).*

### Mediterranean Diet Patterns and the Risk of Lung Cancer

**FIGURE 2:**
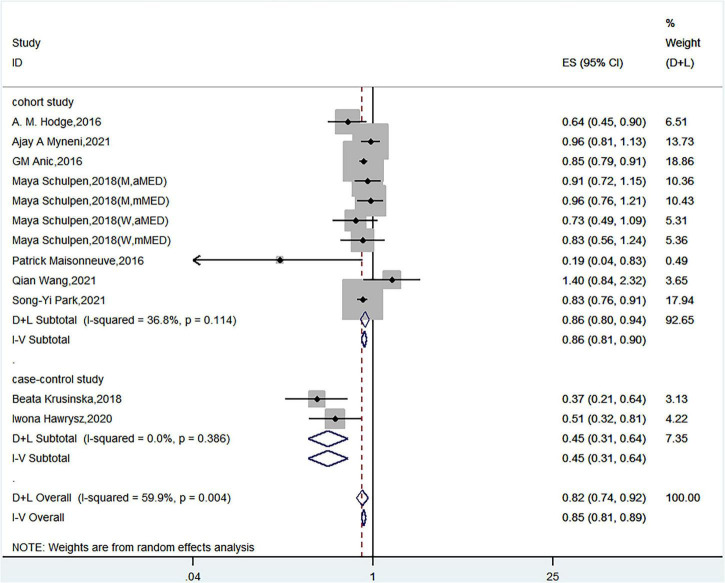
The relationship between Mediterranean diet patterns and the risk of lung cancer.

Nine articles discussed the relationship between Mediterranean diet patterns and the risk of lung cancer. Schulpen et al. used two Mediterranean diet assessment methods and assessed the risk of lung cancer in both men and women (15). Twelve sets of data were therefore used in the meta-analysis. A meta-analysis that used a random-effects model showed a significant negative correlation between Mediterranean diet patterns and the risk of lung cancer (HR = 0.82, 95% CI = 0.74–0.92) with high heterogeneity (*I*^2^ = 59.9%, *P*< 0.05) (Figure 2).

Subgroup analyses on geographic area, study type, number of cases, and adjustments for energy intake, family history, age, ethnicity, and education showed a significant association between Mediterranean diet patterns and the risk of lung cancer. The association was overall consistent in analyses stratified by publication time, MD score system, and adjustments for smoking status (Table 3).

**TABLE 3:** Subgroup analysis of the relationship between Mediterranean diet patterns and the risk of lung cancer.

Subgroups	*N*	RR (95%CI)	Heterogeneity			*P*_–heterogrneity_
			*P-*value	Q statistic	*I* ^2^	
Total	12	0.82 (0.74, 0.92)	0.000	27.47	59.9%	0.004
Geographic area						
Europe	8	0.75 (0.58, 0.97)	0.030	22.50	68.9%	0.002
Non-European	4	0.85 (0.80, 0.89)	0.000	4.89	38.6%	0.180
Study type						
Cohort	10	0.86 (0.81, 0.90)	0.000	14.23	36.8%	0.114
Case-control	2	0.45 (0.31, 0.64)	0.000	0.75	0.0%	0.368
Publication time						
2016	3	0.70 (0.48, 1.02)	0.064	6.16	67.5%	0.046
2018	5	0.78 (0.61, 1.00)	0.047	10.50	61.9%	0.033
After 2020	4	0.87 (0.70, 0.108)	0.201	10.65	71.8%	0.014
No. of cases						
≥1,000	10	0.86 (0.81, 0.90)	0.000	14.23	36.8%	0.114
≤1,000	2	0.45 (0.31, 0.64)	0.000	0.75	0.0%	0.368
Md score system						
AMED	7	0.85 (0.81, 0.90)	0.002	10.32	41.9%	0.112
MMED	2	0.93 (0.79. 1.10)	0.423	0.10	0.0%	0.751
Others	3	0.54 (0.42, 0.69)	0.000	2.75	27.2%	0.253
Adjustments						
Smoking status						
Yes	8	0.79 (0.68, 0.91)	0.001	25.34	72.4%	0.001
No	4	0.89 (0.77, 1.03)	0.117	1.50	0.0%	0.682
Energy intake						
Yes	10	0.86 (0.81, 0.90)	0.000	14.32	36.8%	0.114
No	2	0.45 (0.31, 0.64)	0.000	0.75	0.0%	0.386
Family history						
Yes	5	0.85 (0.79, 0.91)	0.009	2.21	0.0%	0.697
No	7	0.74 (0.59, 0.93)	0.000	25.25	76.2%	0.000
Age						
Yes	6	0.76 (0.64, 0.92)	0.004	20.42	75.5%	0.001
No	6	0.89 (0.80, 0.99)	0.033	5.78	13.4%	0.329
Ethnicity						
Yes	4	0.86 (0.81, 0.90)	0.003	5.91	49.2%	0.116
No	8	0.70 (0.55, 0.87)	0.002	19.61	64.3%	0.006
Education						
Yes	9	0.86 (0.82, 0.90)	0.000	10.45	23.4%	0.235
No	3	0.43 (0.30, 0.61)	0.000	1.91	0.0%	0.384

To explore the source of heterogeneity, we also established a regression model of effect size on a single covariate using the year of publication, design type, and research area as covariates and performed a single-factor meta-regression analysis. The results of the analysis showed that the type of study design was a covariate with a significant impact on the heterogeneity between studies (*P* = 0.016), whereas the publication year and study area did not have a significant impact on the heterogeneity between studies (*P* = 0.486 and *P* = 0.716, respectively). Subsequently, we included the two covariates of study type and region in the meta-regression model and performed meta-regression analysis of multiple covariates. We found that the variance component between the studies decreased from 0.0195 to 0, indicating that 100% could be explained.

### Dose–Response Analysis

**FIGURE 3:**
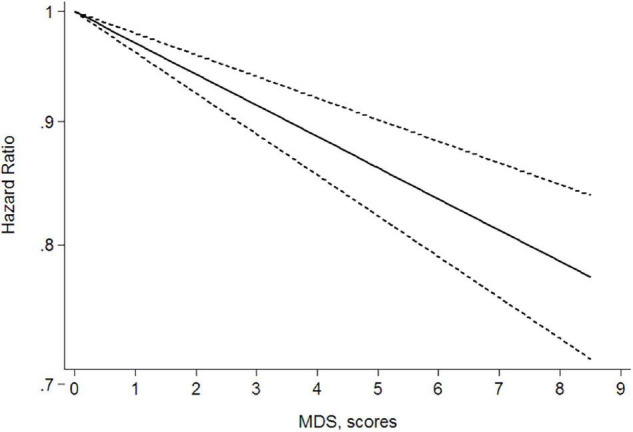
Dose–response metaanalysis of the Mediterranean diet patterns and lung cancer risk.

Given that three of the nine articles did not provide dose grouping and the article by Schulpen et al. (15) included four studies, a total of nine studies (12, 13, 15, 17, 20, 21) were included in the dose–response relationship meta-analysis. The results showed a linear dose–response relationship between Mediterranean diet patterns and the risk of lung cancer and the results were meaningful (*P* < 0.001). In the real life, based on the lowest MDS (0 points), for every three-point increase, the HR for lung cancer = 0.91 and the 95% CI = 0.89–0.94; that is, for every three-point increase in the MDS, the risk of a person developing lung cancer is reduced by 9%. Figure 3 shows the linear dose–response relationship curve. We further explored the dose–response analysis according to the scale range. The linear relation between adherence to a Mediterranean diet pattern and the risk of lung cancer showed a reduction of 6% (RR = 0.94; 95% CI = 0.89–0.99; *P* non-linearity = 0.469) of (0–8) and 10% of (0–9) (RR = 0.90; 95% CI = 0.87–0.93; *P* non-linearity = 0.183) for a three-score increase in adherence to a Mediterranean diet pattern. The relationship was consistent in dose–response analyses stratified by scale range.

### Publication Bias and Sensitivity Analysis

**FIGURE 4:**
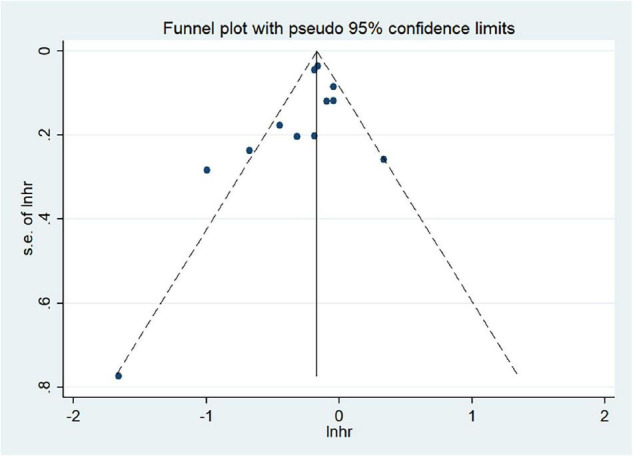
Analysis of publication bias between the Mediterranean diet patterns and lung cancer risk.

**FIGURE 5:**
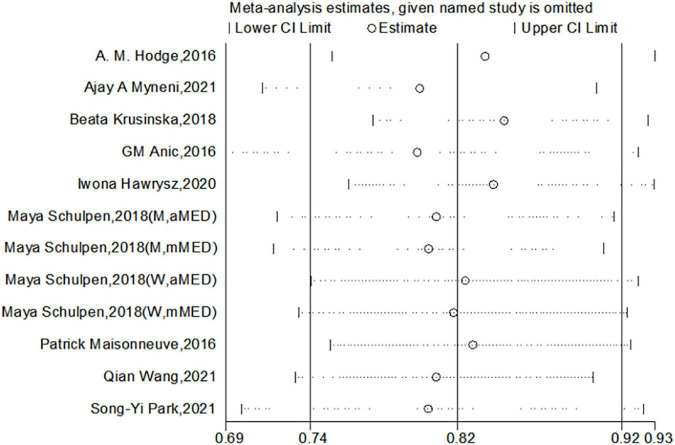
Sensitivity analysis of the Mediterranean diet patterns and the risk of lung cancer.

We used Begg’s regression asymmetry test and Egger’s regression asymmetry test to assess publication bias and found no significant publication bias for either Egger’s test (*P* = 0.226) or Begg’s test (*P* = 0.086) (Figure 4). We conducted a sensitivity analysis to determine whether the meta-analysis results were stable (Figure 5). The results showed that the results did not change very much after excluding each study in turn. The sensitivity was therefore low and the result was relatively stable.

## Discussion

The results of our meta-analysis suggested a significant negative correlation between Mediterranean diet patterns and the risk of lung cancer, thereby showing that Mediterranean diet patterns are a protective factor for lung cancer. Further subgroup analysis showed that the type of study was a possible reason for the heterogeneity of the results. Subsequently, the multivariate meta-regression analysis was performed including the two covariates of study type and region. The results showed that 100% of the sources of heterogeneity could be explained. The results of a meta-analysis of the dose–response relationship showed that based on the lowest MDS (0 points), each three-point increase reduced the risk of lung cancer by 9%. This study is the first meta-analysis to explore the relationship between Mediterranean diet patterns and the risk of lung cancer in the general population. Our findings are of great significance in reducing the incidence of lung cancer.

At present, there are several possible mechanisms linking Mediterranean diet patterns and a reduction in the incidence of lung cancer. First, Mediterranean diets are rich in plant-based foods (e.g., fruit and vegetables), which provide a large amount of dietary fiber and bioactive compounds (e.g., flavonoids, carotenoids, vitamin C, vitamin A, vitamin E, folic acid, and coumarin). A higher intake of dietary fiber can reduce the risk of lung cancer (26). Short-chain fatty acids, fermented from dietary fiber by the gut microbiota, have been shown to benefit host immunity and metabolism in organs including the lungs (27). Animal studies have also found that dietary fiber can reshape the immune environment of the lungs by changing the composition of the lung microbiota (28). The fish and nut products typically found in Mediterranean diets are rich in omega-3 fatty acids and help impede cell proliferation, survival, angiogenesis, and inflammation. Olive oil is used almost exclusively as the dietary oil in Mediterranean diets and contains (-)-Oleocanthal (OC). This compound inhibits the progression of lung cancer and metastasis by targeting c-MET and COX2 (29–31). Red wine, frequently consumed in Mediterranean diets, contains polyphenols that exhibit biological functions, namely, DNA polymerase III gene suppression, T-helper cell facilitation, and DNA methyltransferase and histone deacetylase inhibition (32–35). These effects may slow the transformation and development of cancer cells.

We found a significant negative correlation between Mediterranean diet patterns and the risk of lung cancer. This finding is consistent with the conclusion of a 2003 case–control study, which reported that some typical foods in Mediterranean diets were related to a reduction in the risk of lung cancer (36). Vardavas reported that adhering to a Mediterranean diet pattern and taking in beneficial antioxidants, lipids, and other micronutrients from these diets could have a protective effect against lung cancer and other respiratory diseases. These diets may also have a health impact on smoking and have a positive regulatory role (37), as shown in epidemiological studies. For example, a cohort study conducted by Anic et al. showed that Mediterranean diet patterns are negatively related to lung cancer with HR = 0.85 (0.79–0.91). This association is most common among people who quit smoking, which clearly shows that a Mediterranean diet can reduce the risk of lung cancer in this group (21). In addition, the results of the meta-analysis of the dose–response relationship showed a linear dose–response relationship between the Mediterranean diet and lung cancer. Based on the lowest MDS (0 points), for every three-point increase, the risk of lung cancer was reduced by 9%, indicating that the more food types the population consumes in the Mediterranean diet pattern, the lower the risk of lung cancer. This meta-analysis has important clinical significance and can provide a theoretical basis for preventing lung cancer and improving quality of life.

The heterogeneity among studies is a critical issue in meta-analysis and the existence of heterogeneity directly affects the interpretation of the meta-analysis results. We, therefore, focused on exploring the sources of heterogeneity among studies. Although most of the included research results were adjusted for common influencing factors, such as age, sex, race, and smoking status, other factors among the studies may be a source of heterogeneity. We carried out a subgroup analysis and did not find significant differences in effect between Mediterranean diet patterns and the risk of lung cancer. The results of the meta-regression showed that the type of study design had a significant impact on heterogeneity among studies, whereas the year of publication and study area did not. A total of nine articles, including seven cohort studies and two case–control studies, were included in this meta-analysis because the etiology hypothesis of the case–control study was weaker than that of the cohort study, which led to the heterogeneity. Heterogeneity may also be caused by other factors, such as personal behavior and lifestyle, and environmental and genetic factors.

This study has many advantages. First, the studies included were prospective cohort studies and retrospective case–control studies with large sample sizes. Second, the included studies were all published in the last 5 years and were all of the high quality. The dose–response evaluation of the effects of the included studies may be comprehensive and can effectively explore whether Mediterranean diet patterns are a protective factor for lung cancer. Third, no publication bias was observed in the judgment of *P* detected by the Begg’s method and the funnel chart was symmetrical. This study, therefore, has no publication bias and the sensitivity analysis results were very stable and reliable.

The results of the meta-analysis have scientific value and significance. We found that there was a significant negative correlation between Mediterranean diet patterns and the risk of lung cancer in the general population, which suggests that Mediterranean diet patterns are a protective factor for lung cancer. These results provide clear information for clinical recommendations and the prevention of lung cancer.

This meta-analysis has some limitations. First, most of the included studies were from Europe, with some studies from the United States and Australia. The results may not be universal as a result of the lack of data from Asia and Africa. Second, a stronger association between Mediterranean diet patterns and lung cancer was observed in the case–control study. The inclusion of case–control studies may lead to recall and selection bias. Considering that the retrospective bias in the case–control study and the patient’s early symptoms may cause changes in eating habits, case–control studies may exaggerate the relevance of the merged results in heterogeneity. Third, some studies have pointed out that Mediterranean diet patterns are related to the risk of lung cancer, but data were not reported or the reported data were incomplete and cannot be combined, which may also lead to deviations in the results.

## Conclusion

This meta-analysis showed a significant negative correlation between the Mediterranean diet pattern and the risk of lung cancer. The dose–response relationship showed that based on the lowest MDS (0 points), every three-point increase reduced the risk of lung cancer by 9%. This study is the first meta-analysis to explore the relationship between Mediterranean diet patterns and the risk of lung cancer in the general population. Our findings are of great significance in reducing the incidence of lung cancer. More epidemiological studies, especially cohort studies, should be encouraged to further understand the mechanism. Future studies should be carried out widely in Asia and Africa.

## Data Availability Statement

The original contributions presented in the study are included in the article/Supplementary Material, further inquiries can be directed to the corresponding author/s.

## Author Contributions

HD and TC contributed to the conception, design, search, statistical analyses, and manuscript drafting. XL and TZ contributed to the conception, design, and data interpretation. BL contributed to the conception, design, statistical analyses, and data interpretation. ZL supervised the study. All authors approved the final manuscript before submission.

## Conflict of Interest

The authors declare that the research was conducted in the absence of any commercial or financial relationships that could be construed as a potential conflict of interest.

## Publisher’s Note

All claims expressed in this article are solely those of the authors and do not necessarily represent those of their affiliated organizations, or those of the publisher, the editors and the reviewers. Any product that may be evaluated in this article, or claim that may be made by its manufacturer, is not guaranteed or endorsed by the publisher.
